# [WITHDRAWN] Serum biomarkers of liver fibrosis identify changes in striatal metabolite levels

**DOI:** 10.21203/rs.3.rs-2729490/v2

**Published:** 2024-01-15

## Abstract

The full text of this preprint has been withdrawn by the authors due to author disagreement with the posting of the preprint. Therefore, the authors do not wish this work to be cited as a reference. Questions should be directed to the corresponding author.

